# Laparoscopic Partial Splenectomy for a Giant Congenital Splenic Cyst in a Child: Case Report and Focused Literature Review

**DOI:** 10.1002/ccr3.71870

**Published:** 2026-01-14

**Authors:** Ahmed Alanzi, Dawood Alatefi, Malik Alkabazi, Bano Alsaleh, Khaled M. AlAani, Samah Hakmi

**Affiliations:** ^1^ Anaesthesia and Pain Management Department King Hamad University Hospital Muharraq Bahrain; ^2^ Yemeni‐Syrian Medical Center Ja'ar Abyan Governorate Yemen; ^3^ School of Dentistry Khalij Libya Tripoli Libya; ^4^ Radiology Department King Hamad University Hospital Muharraq Bahrain; ^5^ Faculty of Medicine and Medical Sciences Arabian Gulf University Manama Bahrain

**Keywords:** congenital splenic cyst, giant cyst, laparoscopic partial splenectomy, pediatric surgery, spleen‐preserving surgery

## Abstract

An 11‐year‐old boy with a 16 × 14.5 cm congenital splenic cyst underwent laparoscopic upper‐pole partial splenectomy after negative hydatid workup and vaccination. Recovery was uneventful; histology confirmed epithelial cyst. Spleen‐preserving surgery provided durable symptom relief and preserved function.

## Key Clinical Message

In children with giant congenital splenic cysts, laparoscopic partial splenectomy guided by selective upper‐pole devascularization can relieve symptoms while preserving splenic function. Careful exclusion of hydatid disease, preoperative vaccination, and meticulous hemostasis are key to safe, spleen‐preserving management and low recurrence.

## 1 Introduction

Splenic cysts are rare entities, with an incidence of approximately 0.07%–0.13% in autopsy series [1]. They are broadly classified as parasitic or nonparasitic cysts; nonparasitic cysts are further divided into primary (true) and secondary (pseudocysts) [1]. True cysts possess an epithelial lining and include congenital, neoplastic, and dermoid subtypes, whereas pseudocysts typically develop secondary to trauma, infarction, or infection and lack an epithelial lining [1, 2].

Congenital splenic cysts are extremely uncommon, accounting for only about 10% of nonparasitic splenic cysts [2]. They are believed to arise from embryonic inclusions of peritoneal mesothelial cells or epithelial rests within the splenic capsule. Although most are small and asymptomatic, giant congenital cysts can lead to abdominal pain, distension, or compression of adjacent organs, particularly when they exceed 10–15 cm [2]. In children, splenic cysts are especially uncommon; when present, congenital (epithelial) cysts predominate [3].

Imaging is pivotal for diagnosis, defining size, location, and relations, and for distinguishing congenital epithelial cysts from parasitic cysts, particularly hydatid disease in endemic areas [1].

Historically, total splenectomy was the standard for large or symptomatic splenic cysts. With increasing appreciation of splenic immune function, management has shifted toward spleen‐preserving procedures such as cyst decapsulation/deroofing or partial splenectomy, including laparoscopic approaches [4, 5, 6, 7].

We report the case of a child with a giant congenital splenic cyst managed successfully with laparoscopic partial splenectomy, followed by a focused review of the literature. The objective of this case report is to describe the operative technique in detail and highlight key perioperative considerations including cyst exclusion, preoperative vaccination, and selective upper‐pole devascularization that enable safe spleen preservation in pediatric patients. Additionally, we aim to contextualize this case within the most recent evidence on minimally invasive, spleen‐preserving management of large congenital splenic cysts in children, with emphasis on feasibility, safety, and long‐term outcomes.

## 2 Case Presentation

### 2.1 History and Physical Examination

An 11‐year‐old boy with no known comorbidities presented with a 1‐year history of intermittent back pain radiating to the abdomen, postprandial abdominal heaviness, urinary urgency, and alternating bowel habits. The symptoms had worsened over the previous week. There was no history of trauma, fever, weight loss, or previous abdominal surgery.

On examination, the patient was afebrile and hemodynamically stable. Abdominal inspection revealed fullness of the left upper quadrant without visible peristalsis. Palpation demonstrated splenomegaly extending approximately 10–12 cm below the costal margin, firm in consistency, smooth, and nontender. There were no signs of peritonitis or other systemic findings.

### 2.2 Investigations and Treatment

Initial laboratory investigations, including complete blood count, liver function tests, and coagulation profile, were within normal limits. Echinococcus IgG and confirmatory immunoblot tests were negative (Table 1).

**TABLE 1:** Preoperative Hematology, Biochemistry, Coagulation, and Serology with Reference Intervals.

Test	Result (with units)	Reference range
Hemoglobin	135 g/L	120–160 g/L
White blood cells	7.0 × 10^9^/L	4.0–11.0 × 10^9^/L
Platelets	300 × 10^9^/L	150–450 × 10^9^/L
Sodium	140 mmol/L	135–145 mmol/L
Potassium	4.2 mmol/L	3.5–5.1 mmol/L
Urea (BUN)	4.5 mmol/L	2.5–7.1 mmol/L
Creatinine	50 μmol/L	35–80 μmol/L
Total bilirubin	10 μmol/L	5–21 μmol/L
AST	25 U/L	< 35 U/L
ALT	20 U/L	< 45 U/L
Alkaline phosphatase (ALP)	220 U/L	100–350 U/L
GGT	18 U/L	< 40 U/L
C‐reactive protein (CRP)	2 mg/L	< 5 mg/L
ESR	10 mm/h	< 20 mm/h
PT/INR	12 s/1.0	11–13.5 s/INR 0.8–1.2
aPTT	28 s	25–35 s
Fibrinogen	3.0 g/L	2.0–4.0 g/L
Echinococcus IgG (ELISA)	Negative	Negative
Confirmatory immunoblot	Negative	Negative
Stool ova and parasites	Negative	Negative
Blood culture	No growth	No growth

**FIGURE 1:**
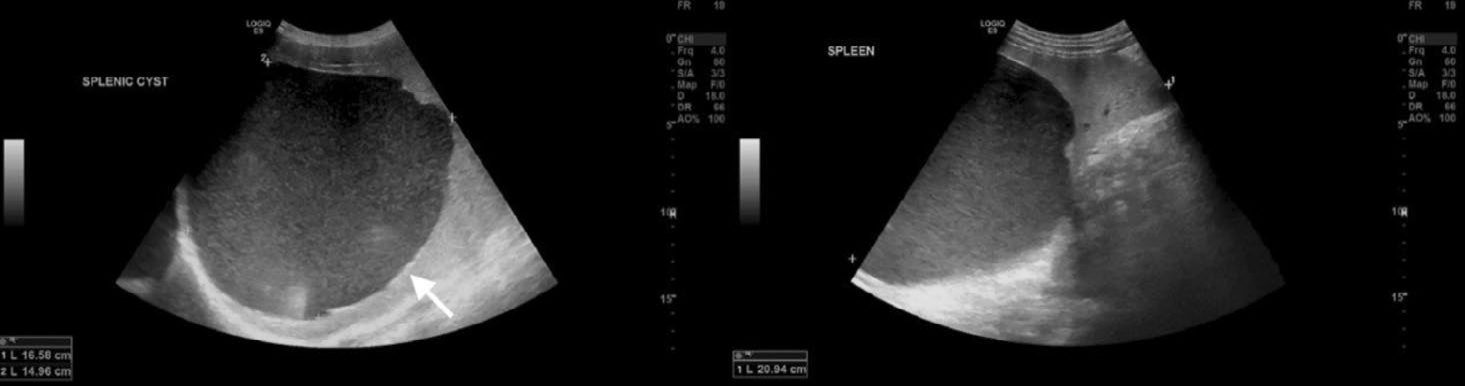
Gray scale ultrasound of the abdomen showing large well defined hypoechoic lesion occupying the upper pole of the spleen, showing internal echoes with posterior acoustic enhancement, few linear hyperechoic discontinues wall lesions suspicious for calcification (a, arrow). No internal vascularity on color Doppler, no soft tissue or fat component.

**FIGURE 2:**
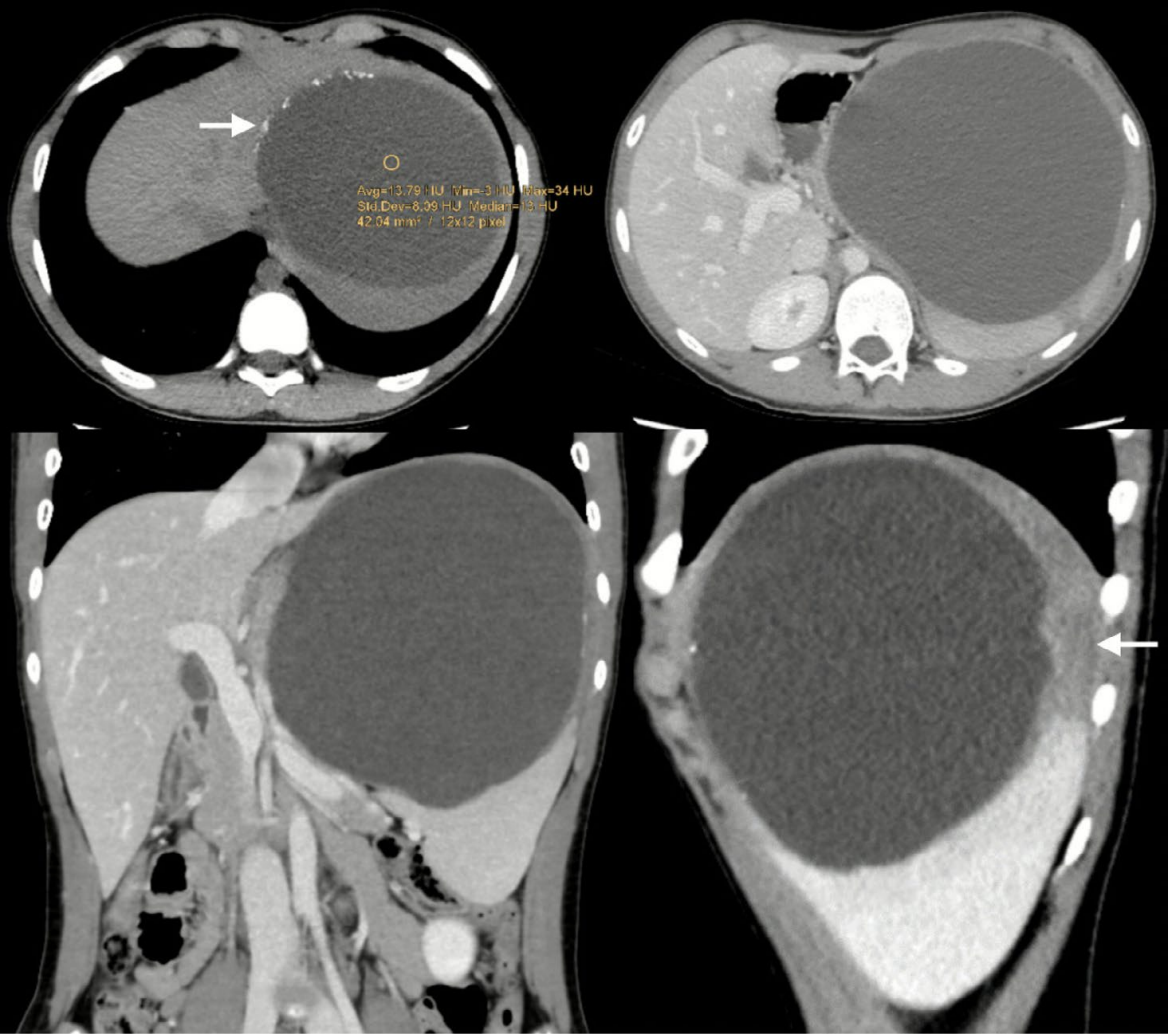
Contrast enhanced CT scan of the upper abdomen showing enlarged spleen with well‐defined hypodense lesion (HU: 14) at the upper pole of the spleen, measuring 13.5 × 14.5 × 16 cm with mass effect upon the adjacent structures. It shows peripheral discontinuous wall calcification (a, arrow); however, no soft tissue or fat component. The upper pole of the spleen is showing hypoenhancement (d, arrow) likely due to edema or infarction.

Abdominal ultrasonography revealed a large, well‐defined cystic lesion arising from the upper pole of the spleen, measuring approximately 16 × 14.5 cm, containing internal echoes suggestive of proteinaceous fluid (Figure 1). Contrast‐enhanced computed tomography (CT) of the abdomen and pelvis confirmed a thin‐walled, unilocular cyst at the upper pole of the spleen with peripheral calcifications and without solid components or septations features consistent with a benign epithelial cyst (Figure 2).

Given the cyst's large size, compressive symptoms, and benign imaging features, surgical management was indicated. After preoperative evaluation by the pediatric hematology team, appropriate vaccinations were administered in accordance with asplenia and hyposplenia protocols.

The patient was positioned in the left lateral decubitus position. Four ports were inserted: a 5 mm infraumbilical (Veress) port, a 12 mm left lumbar port, and two 5 mm ports in the right upper quadrant and epigastric regions. Approximately 1.5 L of straw‐colored fluid was aspirated to decompress the cyst. The upper‐polar branch of the splenic artery was identified and clipped using Hem‐o‐Lok clips, and the resulting avascular demarcation line delineated the resection plane. Adhesions to the stomach, diaphragm, and lateral abdominal wall were carefully divided. The cyst‐bearing upper‐pole segment was resected, and the residual cyst lining adherent to the parenchyma was cauterized to minimize recurrence.

After decompression and devascularization, the splenic flexure of the colon was mobilized, and the short gastric vessels were divided to improve exposure of the upper pole. The upper‐polar arterial branch and corresponding venous tributaries were skeletonized at the hilum before clipping, ensuring a clear boundary between devascularized and viable tissue. Parenchymal transection was performed along this plane using an advanced bipolar energy device, with meticulous stepwise coagulation of small vessels to maintain hemostasis. Dense adhesions between the cyst, diaphragm, and stomach posed a technical challenge and required careful sharp dissection to prevent capsular injury or bleeding. Despite the cyst's large size and distorted anatomy, the procedure was completed laparoscopically without conversion, with an estimated blood loss of approximately 200 mL and no need for transfusion. Hemostasis was secured, and a drain was placed in the splenic bed.

### 2.3 Outcome and Follow‐Up

The postoperative course was uneventful. The patient was mobilized on postoperative day 1, and the drain was removed on day 2 after minimal output. He was discharged on oral antibiotics and analgesics.

**FIGURE 3:**
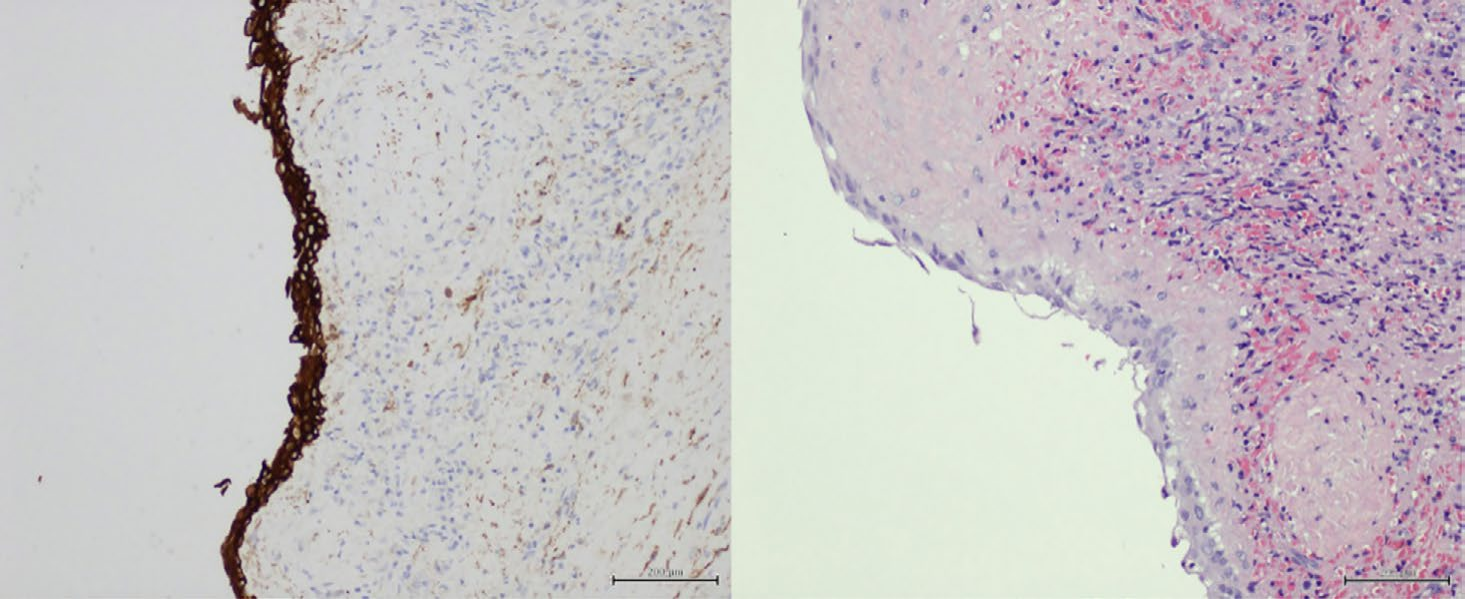
Histopathology specimen of splenectomy and the aspirated fluid shows cyst wall lined by stratified squamous epithelium, confirming the diagnosis of a true epithelial (congenital) splenic cyst and aspirated cyst fluid demonstrated proteinaceous material with no evidence of malignant cells.

Histopathological examination revealed a cyst wall lined by stratified squamous epithelium, confirming the diagnosis of a true epithelial (congenital) splenic cyst. Cytological analysis of the aspirated cyst fluid demonstrated proteinaceous material with no evidence of malignant cells (Figure 3).

**FIGURE 4:**
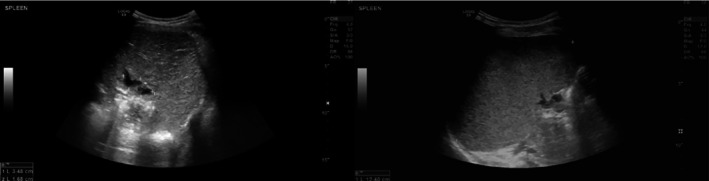
Gray scale ultrasound of the spleen status post operative showing two small located collections in at the site of the operation located at the splenic hilum (a). Follow up imaging showed interval resolution of the previously seen collections with persistent enlargement of the spleen.

At 1‐month follow‐up, the patient was asymptomatic with full resolution of his preoperative abdominal discomfort. Ultrasonography demonstrated preserved splenic tissue with normal vascularity, no residual collections, and no evidence of cyst recurrence (Figure 4). Although long‐term follow‐up is ongoing, the early postoperative course suggests successful splenic preservation and durable symptom relief. Continued clinical and sonographic surveillance is planned to confirm sustained splenic function and monitor for late recurrence.

## 3 Discussion

The first case of a splenic cyst was reported by Andral in 1929 [8]. Since then, the classification of these cysts has significantly evolved. Martin's classification divides splenic cysts into true and false types depending on whether a cellular lining is present in the cyst wall [5]. Secondary or false lesions, also called pseudocysts, lack an epithelial layer and represent nearly 80% of all cases. They typically arise after blunt injury, disrupted hematoma, infarction of splenic tissue, intraparenchymal extension of a pancreatic pseudocyst, or occasionally from a localized abscess. Primary or true cysts, by contrast, show a definite epithelial cover (4). They are thin‐walled, filled with clear to yellow fluid, and often display a trabeculated inner surface with a glistening layer resembling endocardium [9]. These primary cysts typically affect adolescents and young adults during the second to third decade and are much more common in females, although they can occur in children as well [10, 11]. Our patient, an 11‐year‐old boy, thus represents an unusual presentation of a giant congenital splenic cyst in the pediatric male population. Most splenic congenital cysts are solitary, unilocular, and thin‐walled, often lined by squamous or cuboidal epithelium [4]. Because of their epithelial lining and benign nature, they are sometimes called “epidermoid” cysts. Histopathology in our case confirmed a true epithelial cyst lining with Gamna‐Gandy bodies, consistent with the typical description of congenital splenic cysts [4].

Congenital splenic cysts are often asymptomatic when small [12]. In children and adolescents, many cysts are detected incidentally on imaging. However, as these cysts enlarge, they can cause symptoms from mass effect or complications. Our patient had year‐long left upper quadrant discomfort, early satiety, postprandial abdominal heaviness, and even urinary frequency, reflecting compression by the large splenic mass. This correlates with prior reports that large (> 10 cm) cysts can produce vague abdominal or back pain and referred symptoms [4]. Large cyst size often leads to symptoms, including palpable left upper quadrant mass, pain, nausea/vomiting, or respiratory symptoms from diaphragmatic irritation [13, 14]. Furthermore, splenic cysts can lead to splenomegaly, which can cause symptoms such as dyspnea, shoulder pain, and constipation from pressure against adjacent organs [15]. In large congenital cysts, up to 30%–40% present with a painless left upper quadrant mass as the main finding, but our patient was symptomatic [4].

Imaging confirmed a giant simple splenic cyst. Ultrasonography and CT showed a well‐demarcated, thin‐walled, fluid‐filled splenic cyst, with peripheral wall calcifications, classic features of a benign epithelial cyst [1]. No solid components or internal septations were seen. Hydatid infection was excluded as serologic tests such as Echinococcus IgG ELISA were negative. In prior case series, negative hydatid serology plus imaging allows the surgeon to proceed without scolicidal precautions [4]. Our patient underwent preoperative vaccinations for encapsulated organisms in anticipation of any splenectomy. Current guidelines recommend administering pneumococcal, meningococcal, and Haemophilus vaccines at least 2 weeks prior to elective splenectomy or splenic injury [16, 17].

The decision to operate was driven by the patient's symptoms and cyst size. In the literature, most authors agree that asymptomatic, small cysts (often < 4–5 cm) may be managed conservatively with observation [12, 18]. However, larger cysts (> 5–6 cm) carry risks of rupture, hemorrhage, or persistent symptoms, and elective surgery is usually recommended [19, 20]. However, Lena et al., in their study, reported that asymptomatic large cysts that were managed conservatively did not become symptomatic during the study period [21]. Despite this evidence, most authors recommend surgical options in case of large splenic cysts. A systematic review by Aoun et al. reported that surgical intervention is advised when a nonparasitic splenic cyst is symptomatic or at least 50 mm in diameter [22]. Similarly, a recent pediatrics case series suggests surgical treatment for symptomatic cysts or those ≥ 5 cm in children [12]. Our patient's 16‐cm cyst far exceeded this threshold and was clearly symptomatic, meeting the usual criteria for surgery.

Historically, total splenectomy, often via open laparotomy, was the standard for large splenic cysts. However, greater appreciation for splenic immune function has shifted the paradigm toward spleen‐preserving techniques [4, 22]. Recently, several spleen‐conserving approaches are described: laparoscopic or open partial splenectomy, cyst excision with rim of splenic tissue, near‐total cyst unroofing or decapsulation, or fenestration/marsupialization of the cyst wall [22]. In a systematic review by Aoun et al., 83.64% underwent organ‐preserving surgery whereas only 16.36% had total splenectomy [22]. In children, partial splenectomy is often emphasized to preserve some part of spleen tissue and thus maintain immune function [6]. Splenectomy remains an option when cyst location or complexity precludes partial resection [4]. The shift to minimally invasive surgery has extended to splenic cysts. Laparoscopy offers less postoperative pain, shorter hospital stay, quicker recovery, and improved cosmesis compared to open surgery, while still achieving effective cyst treatment [23].

Our surgical strategy and postoperative course align with recent reports of laparoscopic spleen‐preserving cyst treatment. For example, in a pediatric series of benign splenic tumors including cysts, laparoscopic partial splenectomy was performed in 9 of 24 cases with no major complications [23]. That study found no significant differences in operating time or bleeding between laparoscopic total and partial splenectomy groups, supporting the feasibility of laparoscopy even in complex pediatric cases [23]. Keckler et al., in their study, compared open versus laparoscopic splenic cyst excision in children. They reported that the laparoscopy group had a significantly shorter hospital stay (mean 1.6 vs. 2.8 days) and comparable outcomes [23].

Importantly, laparoscopic partial splenectomy appears to be effective at preventing recurrence. Decapsulation or near‐total cyst unroofing has been advocated by some authors to minimize residual cyst lining [4], but partial splenectomy, with complete removal of the involved pole, can achieve similar goals. Our case aligns with accumulating evidence that laparoscopic spleen‐sparing surgery is safe and effective for pediatric congenital splenic cysts. For example, Sadjo et al. reported three children with epidermoid cysts treated by laparoscopic partial splenectomy. All had successful resection with minimal blood loss and no recurrences at up to 32 months follow‐up [6].

For pediatric surgeons, this case underscores several practical points. First, giant congenital splenic cysts in children should prompt careful evaluation for spleen‐preserving options rather than defaulting to total splenectomy. Second, preoperative planning with vaccination, hydatid exclusion in endemic areas, and assessment of segmental vascular anatomy enables safe selective devascularization and partial splenectomy. Third, laparoscopic access is feasible even for very large cysts when performed in a controlled setting with meticulous hemostasis and readiness to convert if necessary. Taken together with the contemporary literature, our experience supports laparoscopic partial splenectomy as a rational first‐line approach for giant, symptomatic congenital splenic cysts in children who have adequate residual parenchyma.

## 4 Literature Review

**FIGURE 5:**
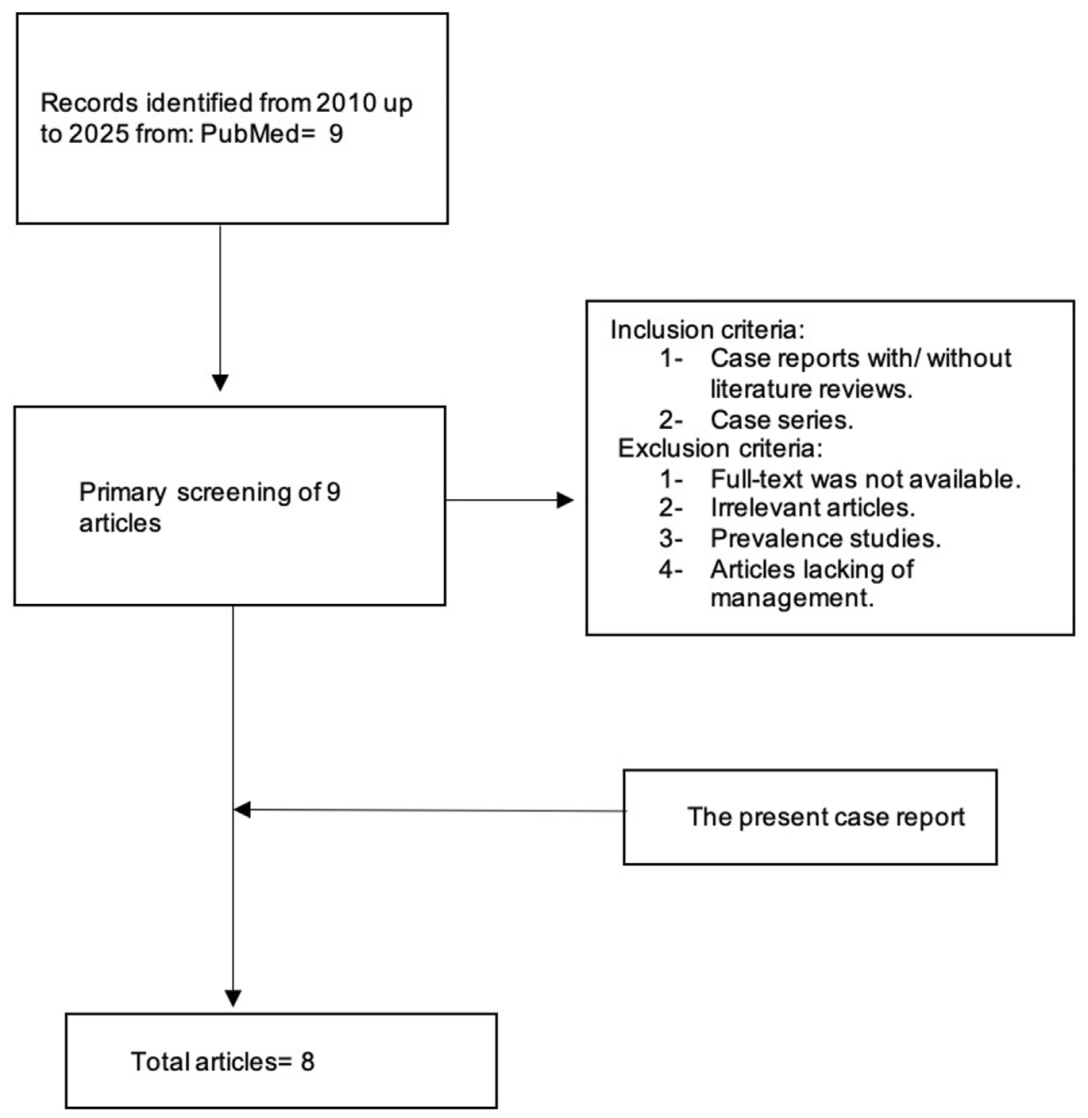
Flowchart of literature search.

A systematic literature search was conducted in PubMed from 2010 to 2025 using the keywords “Giant” AND “Congenital” AND “Splenic” AND “Cyst”. A total of nine articles were initially identified. Eight articles met the eligibility criteria and were included in the review, in addition to the present case report (Figure 5).

Primary nonparasitic splenic cysts are rare clinical entities, with most cases reported in children and young adults. The reviewed literature highlights their variable presentation, ranging from incidental findings to acute abdominal pain, often due to the large size or complications of the cysts.

Across the reported cases, cyst dimensions frequently exceeded 10 cm, with several classified as “giant cysts” (> 15 cm). Clinical manifestations included left upper quadrant pain, abdominal mass, weight loss, and in some cases, pleuritic pain or posttraumatic onset. Laboratory findings were typically unremarkable, with occasional elevations of tumor markers such as CA 19–9, while hydatid serology was consistently negative, helping to exclude parasitic etiologies.

Management strategies varied depending on cyst size, symptoms, and splenic involvement. Splenectomy was chosen in cases of massive cysts, hilar involvement, or suspicion of infection. In contrast, minimally invasive approaches such as laparoscopic decapsulation or cyst resection were successful in preserving splenic tissue, particularly in young patients, with good postoperative outcomes and no recurrence on follow‐up. However, complications such as vascular compromise necessitating completion splenectomy have been reported, underscoring the technical challenges of spleen‐preserving surgery.

In more recent series, minimally invasive, spleen‐preserving techniques have increasingly replaced open total splenectomy, particularly in the pediatric population. Aoun et al. reported that, among patients with giant nonparasitic splenic cysts, over 80% underwent organ‐preserving procedures, with low recurrence rates and acceptable morbidity [22]. Similarly, pediatric reports by Kong et al. and Sadjo et al. demonstrated that laparoscopic partial splenectomy or cyst excision can be safely performed in children while maintaining splenic function and avoiding long‐term infectious risks [6, 12].

Histopathological analysis consistently confirmed congenital epithelial (epidermoid) cysts, often lined by squamous epithelium, occasionally associated with mesothelial elements or inflammatory changes. Long‐term follow‐up in these cases demonstrated favorable outcomes with no malignant transformation.

Overall, the literature emphasizes that while conservative management may be sufficient for small, asymptomatic cysts, surgical intervention is warranted for giant or symptomatic lesions, with spleen‐preserving approaches preferred whenever feasible to maintain immune function (Table 2).

**TABLE 2:** Overview of the included case report.

Study	Country	Sex	Age (years)	Size (cm)	Presentation	Diagnosis	Surgical approach
Coulier (2020) [7]	Belgium	F	19	21.5 × 15 × 21	Progressive subcostal pain of 6 months' duration.	Giant splenic epithelial congenital cyst	Splenectomy with cyst excision
Termos (2020) [4]	Kuwait	F	22	20 × 17 × 15	Left upper‐quadrant and pleuritic pain, food intolerance, and weight loss	Primary nonparasitic splenic cyst	Laparoscopic cyst decapsulation with spleen preservation
Elhardello (2018) [24]	UK	F	19	12.5 × 9.7 × 10.7	Severe left upper abdominal pain	Giant splenic cyst	Laparoscopic decapsulation with ~90% cyst wall excised and the lining cauterized
Tassopoulos (2017) [25]	USA	F	6	7.1 × 6.2 × 6	Worsening abdominal pain after blunt trauma	Giant congenital splenic cyst presenting as peritonitis	Elective laparoscopic cyst resection
Esposito (2014) [26]	Italy	M	9	20 × 13 × 21	Acute left upper quadrant pain and a palpable abdominal mass	Giant epidermoid splenic cyst	Open splenectomy was performed due to cyst size, hilar location, and residual atrophic splenic tissue.
Pitiakoudis (2011) [19]	Greece	F	19	17 × 12 × 15.5	Upper quadrant pain and a palpable mass	Giant epidermoid splenic cyst	Laparoscopic partial cyst wall resection with splenic preservation, but subsequent imaging suggested vascular compromise, leading to completion splenectomy.
Shukla (2010) [27]	India	M	10	15 × 13	Gradually enlarging left hypochondrial mass over 7 months, assocaited with pain and intermittent fever	Giant congenital infected splenic cyst	Laparotomy, 1.5 L of brown fluid was aspirated, and total splenectomy was performed due to cyst size and hilar involvement

## 5 Conclusion

Congenital splenic cysts are rare in children and may reach considerable size before becoming symptomatic. Accurate diagnosis, exclusion of parasitic disease, and thoughtful surgical planning are essential to optimize management. Laparoscopic partial splenectomy offers a minimally invasive, spleen‐preserving approach that allows complete removal of the cyst lining while maintaining splenic function. This case adds to the growing evidence that partial splenectomy is an effective option for giant congenital splenic cysts, providing reliable symptom relief and a low risk of recurrence.

## Author Contributions

**Ahmed Alanzi:** conceptualization, data curation, investigation, supervision, writing – original draft, writing – review and editing. **Dawood Alatefi:** investigation, methodology, software, validation, writing – original draft, writing – review and editing. **Malik Alkabazi:** software, validation, writing – original draft, writing – review and editing. **Bano Alsaleh:** investigation, resources, validation. **Khaled M. AlAani:** investigation, resources, validation. **Samah Hakmi:** investigation, resources, validation.

## Funding

The authors have nothing to report.

## Consent

Formal written informed consent for publication of this case report was obtained from the parents and it will be available upon request by the journal chief editor.

## Conflicts of Interest

The authors declare no conflicts of interest.

## Data Availability

The data used to support the findings of this study are included in the article.
